# The cultivation of *Panax notoginseng* enhances the metabolites and microbial network complexity in the soil of *Pinus armandii* rather than *Pinus kesiya*

**DOI:** 10.3389/fmicb.2025.1616266

**Published:** 2025-08-06

**Authors:** Jingying Hei, Yue Li, Rui Rui, Noor Faisal, Jiansong Peng, Biao Wang, Shu Wang, Xiahong He

**Affiliations:** ^1^College of Landscape Architecture and Horticulture, Southwest Forestry University, Kunming, China; ^2^Department of Biochemistry and Molecular Biology, School of Life Sciences, China Medical University, Shenyang, China; ^3^Yunnan Provincial Key Laboratory for Conservation and Utilization of In-forest Resource, Southwest Forestry University, Kunming, China

**Keywords:** Sanqi cultivation, microbial community, differential metabolites, network complexity, network stability

## Abstract

**Introduction:**

The species of tree most appropriate for the cultivation of Sanqi in an understory environment is pine. Nevertheless, the precise type of pine that confers the greatest benefit to soil health during Sanqi cultivation has not been definitively established.

**Methods:**

Herein, four distinct land use configurations were established, including the *Pinus armandii*, *Pinus kesiya*, Sanqi–*Pinus armandii* (SPA), and Sanqi–*Pinus kesiya* (SPK) systems. High-throughput sequencing technology and metabolomics analysis were used to comparatively evaluate variations in bacterial and fungal community structures and soil metabolites between the SPA and SPK systems.

**Results and discussion:**

After cultivating Sanqi, the content of total phosphorus, ammonium nitrogen, and total potassium as well as water content and soil pH were significantly increased in *P. armandii* soil. Moreover, the bacterial and fungal copy numbers, alpha- and beta-diversity, remained unchanged in the soil of *P. armandii*, but significantly decreased in the soil of *P. kesiya* following Sanqi planting. Moreover, Sanqi cultivation significant increased complexity of the microbial network in *P. armandii* rather than *P. kesiya* soil, while the network stability was maintained. Structural equation modeling indicated that soil enzymes, metabolites, and edaphic factors enhanced the complexity of the microbial network in *P. armandii* soil in SPA system. Additionally, the content of eight differentially accumulated metabolites (DAMs) was significantly increased in the rhizosphere and bulk soils of *P. armandii*. In conclusion, the cultivation of Sanqi benefits the microbiome and metabolites in *P. armandii* rather than *P. kesiya* soil, thus providing an important theoretical foundation for the sustainable development of Sanqi cultivation.

## Graphical abstract

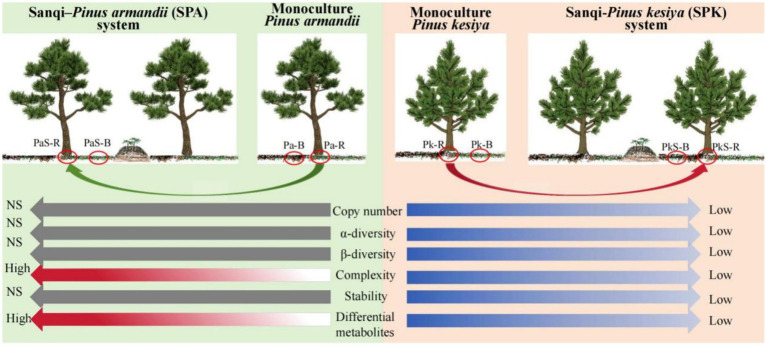

## Highlights

- After cultivating Sanqi, bacterial and fungal copy numbers, *α*- and *β*- diversity were unchanged in *P. armandii* soil but significantly decreased in *P. kesiya* soil.
- Sanqi cultivation increased microbial network complexity in *P. armandii* rather than *P. kesiya* soil, while maintaining network stability.
- Differential metabolites increased significantly in the rhizosphere and bulk soil of *P. armandii* after Sanqi planting.
- After cultivating Sanqi, soil enzymes, metabolites, and edaphic factors increased the complexity of the microbial network in *P. armandii* soil.

## 1 Introduction

Sanqi (*Panax notoginseng*) is a perennial herbaceous plant that belongs to the family Araliaceae. The practice of artificial cultivation of Sanqi began in China four centuries ago, with Yunnan and Guangxi Provinces being the primary areas of cultivation (Chen et al., 2022). Sanqi has considerable therapeutic effects such as promoting blood circulation and resolving blood stasis, reducing swelling, and relieving pain (Hei et al., 2023). Furthermore, its root and flowers are key ingredients of several Chinese patent medicines (Li et al., 2024). A conventional management of Sanqi cultivation is associated with continuous cropping obstacles of considerable severity, with a 7–10-year interval typically required before replantation can be done (Tan et al., 2017). Conversely, Sanqi cultivation in the forest understory under organic management is associated with several benefits, including improvements in the quality of Sanqi (Li et al., 2024), mitigation of continuous cropping obstacles (Hei et al., 2023, 2024), enhancement of soil microbial diversity (Jia et al., 2022), alleviation of carbon limitation in pine soils (Rui et al., 2025), and increase in the content of differentially accumulated metabolites (DAMs) (He et al., 2025). Previous studies have revealed that suitable forest tree species for Sanqi cultivation include broadleaf (with trees such as walnut; Gong et al., 2016), coniferous (*Pinus armandii*, *Pinus kesiya*, and *Pinus yunnanensis*; Jia et al., 2022), and mixed (a combination of coniferous and broadleaf trees; Deng et al., 2020) forests. However, previous research has indicated that Sanqi cultivated under the pine trees can achieve better quality and higher yield (Shi et al., 2021). Therefore, the Sanqi–pine agroforestry system subjected to organic management has been widely promoted in Yunnan and covers an area of 666.67 hectares (Hei et al., 2023). Sanqi is a key plant species in the agroforestry system, and previous research on Sanqi has chiefly focused on the optimization of planting density (Liu et al., 2021), prevention and control of pests and diseases (Luo et al., 2022; Wang et al., 2023), quality analysis (Chen et al., 2022; Hei et al., 2023), identification of metabolites (Shi et al., 2022), and prevention as well as control of continuous cropping obstacles. Nonetheless, it is imperative to consider the sustainable development of both plant species within the agroforestry system. Consequently, undertaking research on the impact of Sanqi cultivation on soil health and its impact on the growth of pine trees is expected to aid the establishment of a robust theoretical foundation for the sustainable development of the Sanqi–pine agroforestry system.

Soil microbes are considered a key indicator of soil health in terrestrial ecosystems (Wilhelm et al., 2022), and they are significantly influenced by cropping patterns and ecological niches (Karoline and Jeroen, 2012; Qin et al., 2017; Wang et al., 2023). The conversion of land use pattern to agroforestry systems has had a significant impact on the abundance and community structure of soil microbes (Araujo et al., 2012; Beule et al., 2022). For example, the walnut–tea agroforestry system has been shown to notably enhance the abundance and structural composition of bacterial and fungal communities in the soil (Bai et al., 2022). Likewise, the mulberry–peanut agroforestry system has been observed to augment the diversity and richness of bacterial and fungal populations (Li M. N. et al., 2022). Conversely, a decline in the richness and diversity of soil microbes has been observed in other systems, including the ginkgo–metasequoia and ginkgo–rubber agroforestry systems (Wang et al., 2017; Guo et al., 2021). These varying outcomes may be attributed to a confluence of factors encompassing plant species (Mortimer et al., 2015) and growth environments (Guo et al., 2021). Furthermore, the conversion of pine forests into Sanqi–pine agroforestry systems facilitates the transfer of beneficial microbial communities from Sanqi to the pine trees, subsequently inducing an elevation in the alpha (*α*)–diversity of fungi in the soil where the pine trees grow. Conversely, the transfer of microbes from pine trees to Sanqi influences the microbial composition and endophytes associated with Sanqi (Jia et al., 2022). The cultivation of Sanqi has been shown to result in higher diversity of fungi compared to that of bacteria in the rhizosphere of pine trees, while the bacterial and fungal diversity in the soil of Sanqi remain consistent. Additionally, enhancements have been observed in the diversity, community structure, network complexity, and stability of carbon-fixing bacteria in the soil in which Sanqi and pine were grown (He et al., 2025), as well as in the diversity and network complexity of nitrogen-fixing bacteria in the soil in which Sanqi was cultivated (Zhao et al., 2025). This phenomenon is attributed to the differences in the types of root exudates produced by various plants (Ding et al., 2022) and the unique environmental conditions in the soil (Jiang et al., 2023).

Soil metabolites serve as biomarkers for changes in the community composition of soil microbes (Li et al., 2023), and the metabolite components are significantly influenced by different cultivation practices and ecological niches. The primary soil metabolites in pine forests include organic and phenolic acids (Shao et al., 2011). However, the cultivation of Sanqi in the forest understory leads to an increase in the content of organic acids such as phthalic and palmitic acids in the soil (Hei et al., 2023). An increase in the concentration of these organic acids beyond 150 mg/kg negatively affects plant growth, soil environment, and the interactions among the members of the microbial communities (Hei et al., 2023, 2024). This is attributed to the transport capacity of organic acids, despite the differences in the transport range among different plants. For instance, 3,4-dihydroxybenzoic acid and vanillin cause significant autotoxicity within a 20-cm range of the *Rehmannia glutinosa* root system, and this effect weakens with increasing distance from the root system (Zhang et al., 2016). By contrast, benzoic acid shows strong allelopathic activity within a 6–10-cm zone of the *Panax ginseng* root system (Ren, 2016). On the one hand, organic acids can regulate soil metabolites via interactions with other organic and phenolic acids (Shen et al., 2020; Hei et al., 2024). Conversely, they influence the structure of microbial communities and thereby, indirectly affect soil metabolites (Zhou et al., 2023). Furthermore, ecological niches (rhizosphere and bulk) have a significant impact on soil metabolites (Zhalnina et al., 2018). Studies have shown that metabolites in the rhizosphere soil exhibit trends of increase (Song et al., 2020), decrease (Sun et al., 2022), or lack of significant changes (Chen et al., 2018) compared to bulk soil. These variations are attributed to differences in plant root morphology (Iannucci et al., 2021), root exudates (Bi et al., 2021; Liu et al., 2023), soil factors (Tian et al., 2022), and microbial community structure (Cheng et al., 2022). In summary, the soil metabolites released from one plant may exert either positive or negative effects on the growth of other plants in the environment (Wang et al., 2021).

Sanqi thrives in the forests of *P. armandii*, *P. kesiya*, and *P. yunnanensis* understorey. Among these three pine species, the most extensive areas suitable for Sanqi cultivation are those of *P. armandii* and *P. kesiya*. Previous research has indicated that the microbiomes associated with Sanqi significantly influence the microbial communities related to *P. armandii* and *P. kesiya* (Jia et al., 2022). However, it remains unclear whether Sanqi cultivation affects the metabolites in pine soil. Additionally, microbiomes are closely linked to metabolites, and the metabolites of some medicinal plants can disperse over a distance (Zhang et al., 2016). Thus, four land use patterns, encompassing the *P. armandii*, *P. kesiya*, Sanqi–*P. armandii* (SPA), and Sanqi–*P. kesiya* (SPK) systems, were established. Bacterial and fungal communities, along with soil metabolites, were comparatively analyzed using high-throughput sequencing and LC–MS metabolomics approaches to explore the effects of Sanqi cultivation on the relationship between microbiomes and metabolites. The purpose of this study was: (1) To determine the effects of Sanqi cultivation on the soil microbiomes and metabolites of *P. armandii* and *P. kesiya*; (2) to investigate the changes in the relationship between soil microbiomes and metabolites.

## 2 Materials and methods

### 2.1 Study sites and Sanqi transplantation

The sites corresponding to the SPA and SPK systems are located in Lancang Lahu Autonomous County, Pu’er City, Yunnan Province (with coordinates 22.74°N and 99.82°E, elevation 1457.39 m, average annual temperature 19.2°C, and average annual precipitation 1008.6 mm) and Dadi Water Village, Xundian Hui Autonomous County, Kunming City (coordinates 25.47°N and 103.21°E, elevation 2247.81 m, average annual temperature 15.5°C, and average annual precipitation 1624.0 mm), respectively.

Plots under the SPA and SPK systems with slopes ranging from 5° to 15° were selected in December 2018. After the clearing of stones and vegetation from the soil surface, the soil was plowed to a depth of 20–30 cm, and its pH was adjusted using hydrated lime. A ridge of the following dimensions was constructed along the isoheight of each forest: 40-cm height with 120- and 80-cm width at the base and top, respectively. Subsequently, one-year-old Sanqi seedlings were transplanted in the ridges at a depth of 3–5 cm and with row spacing of (10–15) cm × (10–15) cm; they were then covered with 2–5 cm of soil. Following Sanqi transplantation, the surface of the soil was covered with pine needles of 3–5 cm thickness. The cultivation techniques and routine management practices for Sanqi in the forest understory were carried out as detailed previously (Hei et al., 2023, 2024).

### 2.2 Experimental design and soil sampling

To compare the effects of Sanqi plantation on the soils of *P. armandii* and *P. kesiya*, 24 plots each measuring 10 m × 10 m were established herein, including those with *P. armandii* and *P. kesiya* monocultures and SPA as well as SPK systems; the corresponding soils in these systems are hereafter referred to as Pa-R, Pa-B, Pk-R, Pk-B, PaS-R, PaS-B, PkS-R and PkS-B.

In November 2021, prior to Sanqi harvest, the rhizosphere and bulk soil from each plot were collected using a five-point method (Jia et al., 2024). The soil (0–2 mm) adhering to the root surfaces of *P. armandii* and *P. kesiya* was designated as rhizosphere soil, whereas the soil obtained at a depth of 0–20 cm and distance of 20 cm away from *P. armandii* and *P. kesiya* was considered bulk soil. A total of 24 soil samples were collected, specifically: 8 treatments × 3 replicates. All the soil samples were subsequently combined to form a composite sample, which was treated as a single replicate. The soil samples were passed through a 4-mm sieve and then segregated into three distinct portions that were subsequently preserved at 4°C and −80°C or air-dried in an indoor setting for subsequent analysis.

### 2.3 Analyses of physicochemical characteristics and multiple ecosystem functions

Soil water content (WC) was determined by subjecting the soil samples to drying at 100°C for 24 h. Soil pH was determined using a 1: 5 (w: v) soil slurry. The content of total phosphorus (TP), total nitrogen (TN), nitrate nitrogen (NO_3_^−^–N), and ammonium nitrogen (NH_4_^+^–N) in the soil matrix was measured using a continuous flow analyzer (Seal Auto Analyzer AA3, Germany). Total potassium (TK) was quantified employing a flame atomic absorption spectrophotometer (AA-6300C). The soil organic carbon (SOC) content was determined using the potassium dichromate oxidation method.

### 2.4 DNA extraction and qPCR analysis

The Power Soil DNA Isolation Kit (MoBio, USA) was utilized for extracting DNA from the soil samples (0.5 g). The quality and concentration of the isolated DNA were assessed using agarose gel (1%) electrophoresis and NanoDrop2000 spectrophotometer (Thermo Fisher Scientific, USA), respectively. Absolute quantification of bacteria (16S rRNA) and fungi (ITS1) in the soil samples was performed using the LightCycler^®^480 II system (Roche, Switzerland). The sequences of the primers employed for the quantification of bacteria (338F/806R) and fungi (ITS1F/ITS2R), along with the protocols for qPCR, are presented in Supplementary Table S1. After determining the concentration of the plasmid DNA isolated using a plasmid extraction kit (Takara, China), a standard curve was generated by carrying out qPCR with 10-fold serial dilutions of the plasmid DNA. The amplification efficiencies of the 16S rRNA and ITS genes ranged from 95 to 103%, with R^2^ > 0.99. The Cp value for each sample was ascertained via comparison with the standard curve based on the initial copy number of the 16S rRNA and ITS genes, with three replicates employed for each sample.

### 2.5 Illumina sequencing

High-throughput sequencing of the purified amplicons was conducted at Majorbio Bio-Pharm Technology Company in Shanghai (China) utilizing the Illumina MiSeq PE300 platform (Illumina, USA). The sequence similarity threshold for operational taxonomic units was set at 0.97. The sequences associated with accession numbers PRJNA821648 (bacteria) and PRJNA821834 (fungi) have been archived in the NCBI Sequence Read Archive.

### 2.6 Metabolite profiling using rhizosphere soil samples

Samples of rhizosphere soil (1 g each) from Pa, Pk, PaS, and PkS were accurately weighed and mixed with 1 mL of extraction solution (4:1 [v/v] mixture of methanol: water). The samples were pulverized in a frozen tissue grinder at −10°C and 50 Hz, followed by incubation at −20°C for 30 min. After centrifuging for 15 min at 13,000 rpm and 4°C, 120 μL of a 1:1 (v/v) acetonitrile mixture: water was added, and the sample was extracted by ultrasonication at a low temperature (5°C, 40 kHz) for 5 min. Subsequently, the sample was subjected to another centrifugation step for 15 min. The supernatant was transferred to sample vials for subsequent liquid chromatography–mass spectrometry analysis (Thermo Scientific, USA). The data were processed and annotated using Progenesis QI (Waters Corporation, USA) and various databases (HMDB, KEGG) for metabolite identification.

### 2.7 Statistical analysis

The sample data adhered to the assumptions of homogeneity and normal distribution, as evidenced by the Levene’s test (*p* > 0.05) and the Shapiro–Wilk test (p > 0.05). Then, the measured edaphic factors and microbial abundances were subjected to one-way analysis of variance utilizing SPSS 23 (SPSS Inc., USA). The *α*- (Chao and Shannon indices) and beta (*β*)–diversity were calculated using QIIME and the Bray–Curtis distance matrix, respectively. Principal Coordinates Analysis (PCoA) was executed using the vegan package (version 2.5–3). Microbial composition was evaluated using Circos software. The influence of soil characteristics on the microbial community was evaluated using Redundancy Analysis (RDA) (Hei et al., 2023). Before conducting the RDA analysis, and considering that the length of the first axis of the Detrended Correspondence Analysis (DCA) was less than 3, we determined that RDA is more suitable than CCA for analyzing soil characteristics and microbial communities (correlation coefficient > 0.7; Li N. et al., 2022). The topological coefficients were determined using the “igraph” R package to evaluate the network complexity. The greater the node, edge, and graph density, as well as the average degree and clustering coefficient, the lower the average path length and graph diameter, indicating a higher complexity of the network (Xu et al., 2023). We selected the top 200 OTUs with higher abundance to calculate the network stability of bacteria and fungi. Specifically, the network stability analysis was conducted using R software (version 4.2.2), and was measured by the remaining proportion of nodes (proportion of remaining nodes in the network after random removal some nodes) and robustness (proportion of remaining nodes in the network after random deletion of 50% of the nodes) (Wu et al., 2021). Generally, networks with a higher proportion of remaining nodes and robustness exhibit greater stability, with each error bar representing the standard deviation of 100 repeated simulations (Xu et al., 2023). Structural equation modeling (SEM) was employed for investigating the interconnections between *α*-diversity, soil edaphic factors, microbial abundance, soil enzyme activity, microbial community composition, soil metabolites, and network complexity as well as stability. The appropriateness of the model was evaluated using the goodness of fit index (>0.7), as proposed by Rosseel (2012).

## 3 Results

### 3.1 Analysis of the physicochemical properties of various soil samples

Significant increase in the content of TP, NH_4_^+^–N, WC, and TK as well as soil pH was observed in the PaS soil compared to that in the Pa soil, while the content of SOC and TN exhibited a significant decrease. Furthermore, all the assessed indicators in the PaS soil, except for WC and the content of TP and NH_4_^+^–N, exhibited higher concentrations in the rhizosphere soil compared to that in the bulk soil. Additionally, the content of SOC and NO_3_^−^–N was significantly higher in the PkS soil compared to that in the Pk soil, while the content of TN, NH_4_^+^–N, and TK as well as soil pH were significantly lower. Moreover, all the evaluated indicators in the PkS soil, except for WC and the content of SOC and TK, were higher in the rhizosphere soil compared to that in the bulk soil (Supplementary Table S2).

### 3.2 Variations in the copy number and α- as well as *β*-diversity of soil microbes

**Figure 1:**
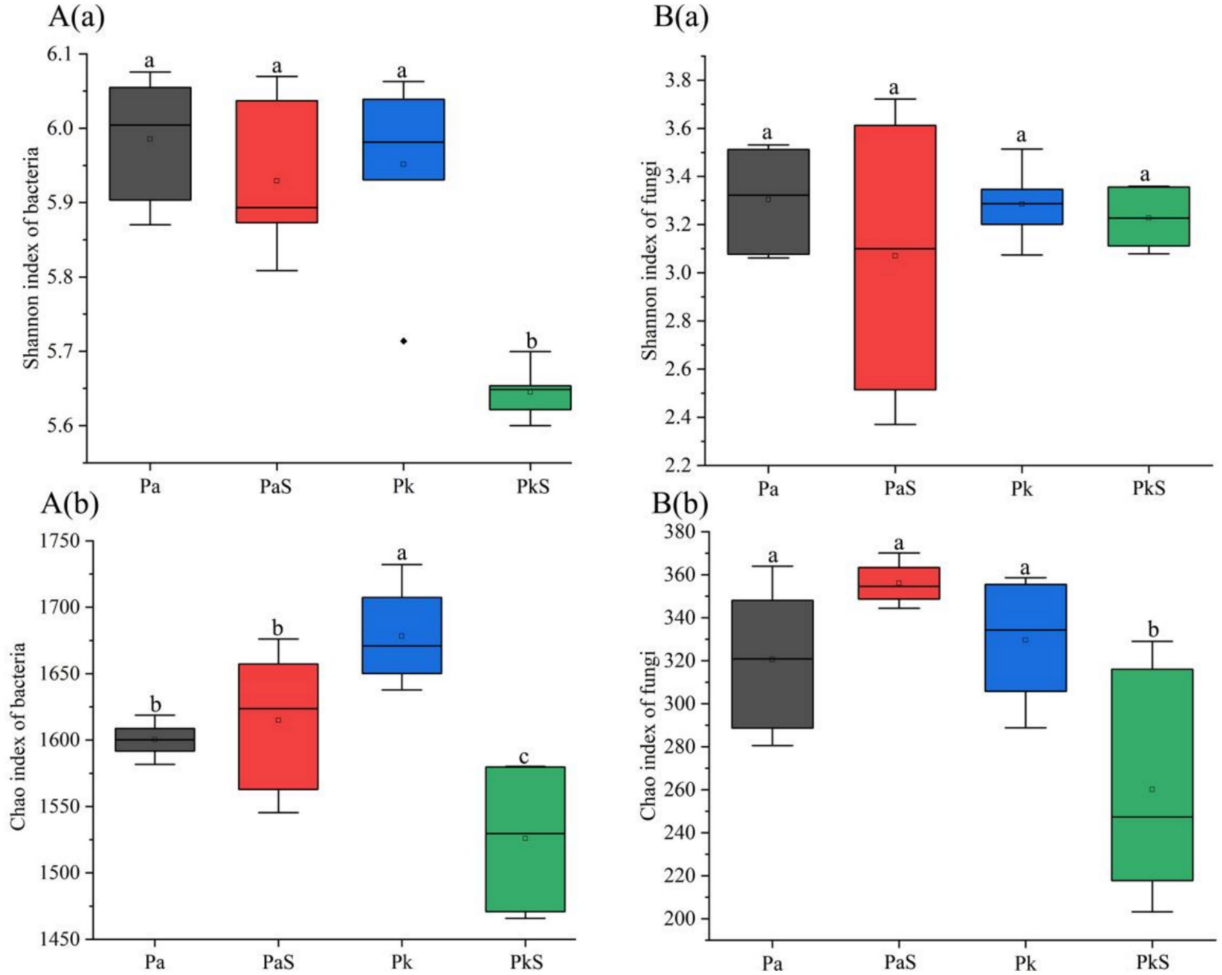
Shannon (a) and Chao (b) indices of bacteria **(A)** and fungi **(B)** under the various land use systems. a, b, and c indicate significant differences at *p* < 0.05, respectively.

The copy numbers and α-diversity of bacteria and fungi, as evaluated using Shannon and Chao indices, were not significantly different in the PaS soil compared to that in the Pa soil. Moreover, the copy numbers of bacteria and fungi were highest in the rhizosphere soil; however, the α-diversity of bacteria and fungi did not significantly differ between the rhizosphere and bulk soils. In general, the copy numbers and α-diversity of bacteria and fungi were significantly reduced in the PkS soil compared to that in the Pk soil. Moreover, the highest values of these indicators were obtained in the rhizosphere soil (Figure 1; Supplementary Figure S1; Supplementary Table S3).

### 3.3 Bacterial and fungal community structures

**Figure 2:**
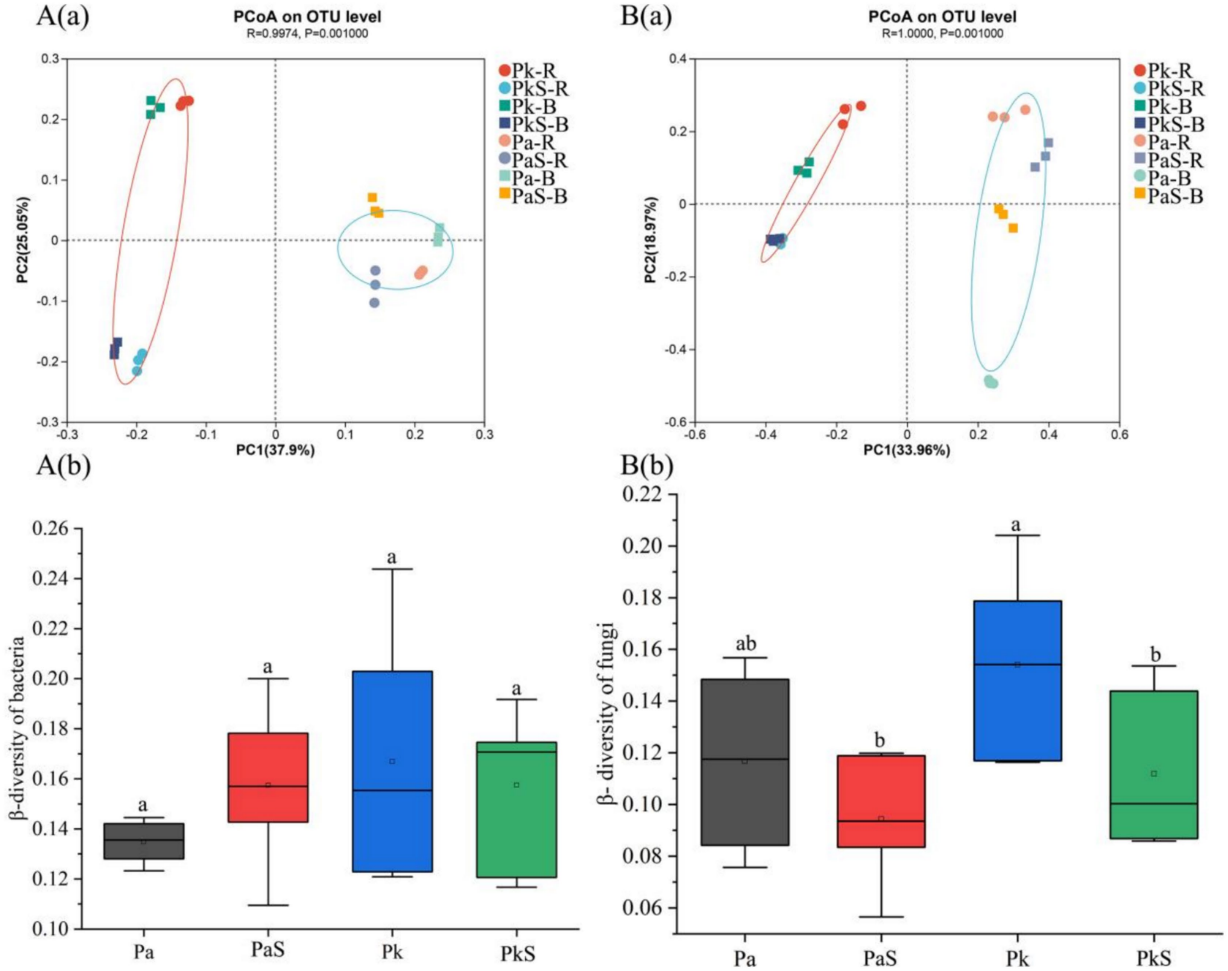
Community structures of soil bacteria **(A)** and fungi **(B)** in the various land use systems. a and b indicate significant differences at *p* < 0.05, respectively.

The results of PCoA indicate that the differences in bacterial (r = 0.9974, *p* = 0.001) and fungal (r = 0.9974, *p* = 0.001) communities among different treatments were both highly significant (Figure 2). ANOSIM analysis based on Bray-Curtis revealed that the influence of tree species on the community structures of bacteria (Bray-Curtis ANOSIM = 0.329, *p* = 0.004) and fungi (Bray-Curtis ANOSIM = 0.418, *p* = 0.007) was greater than that of Sanqi introduction on the community structures of bacteria (Bray-Curtis ANOSIM = 0.112, *p* = 0.04) and fungi (Bray-Curtis ANOSIM = 0.197, *p* = 0.02).

There was no significant difference in the community structure of bacteria (Bray-Curtis ANOSIM = 0.01, *p* = 0.78) and fungi (Bray-Curtis ANOSIM = 0.02, *p* = 0.56) between the rhizosphere and bulk soil. Additionally, greater changes were observed in the community structure of fungi (R = 1.0) than that of bacteria (Supplementary Table S4). Moreover, the values of *β*-diversity of both bacteria and fungi were not significantly different in the PaS soil (Figure 2), mirroring a similar absence of significant variation between the rhizosphere and bulk soils. In the case of PkS soil, the β-diversity of bacteria remained stable whereas that of fungi experienced a significant decline. Nonetheless, significant differences were not observed between the rhizosphere and bulk soils (Supplementary Table S5).

**Figure 3:**
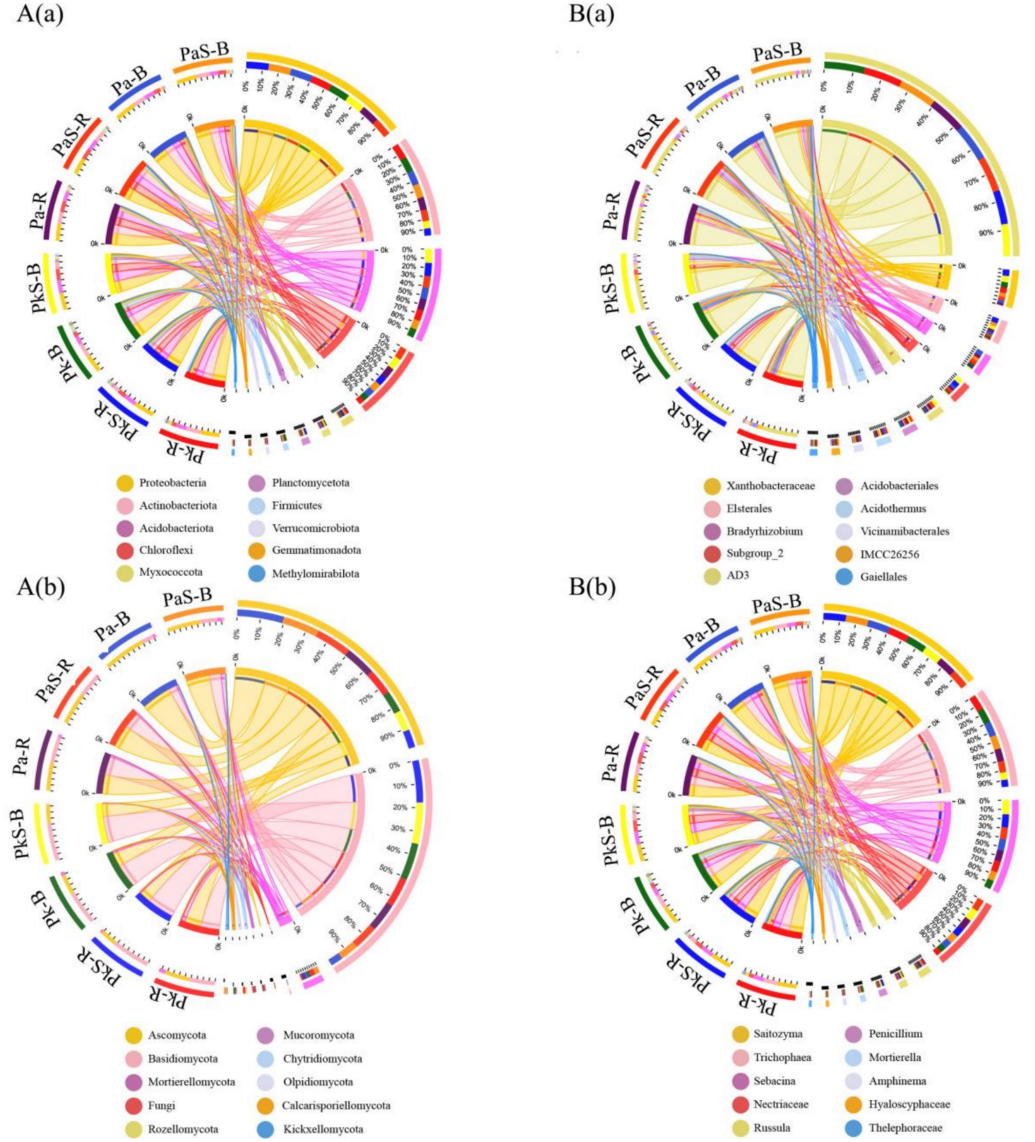
Relative abundance of bacteria **(A)** and fungi **(B)** at the level of (a) phylum and (b) genus.

The dominant bacterial phyla included Proteobacteria (32.88%), Actinobacteria (19.95%), Acidobacteriota (18.68%), and Chloroflexi (14.84%), whereas the dominant fungal phyla were Ascomycota (47.06%), Basidiomycota (46.68%), and Mortierellomycota (7.72%; Figure 3). Moreover, the cultivation of Sanqi increased the abundance of Chloroflexi in PaS and soils while decreasing that of Actinobacteria. Additionally, the abundance of Ascomycota and Mortierellomycota increased significantly in the PaS but not PkS soil, whereas that of Proteobacteria, Acidobacteriota, and Basidiomycota decreased significantly (Figure 3).

A closer look revealed that the dominant bacteria belonged to *Xanthobacteraceae* (7.92%), *Elsterales* (5.30%), *Bradyrhizobium* (5.00%), *Subgroup_2* (4.74%), *AD3* (3.94%), *Acidobacteriales* (3.58%), *Acidothermus* (3.53%), *Vicinamibacterales* (2.56%), *IMCC26256* (2.38%), and *Gaiellales* (2.24%). Similarly, the dominant fungi belonged to *Saitozyma* (11.41%), *Trichophaea* (10.30%), *Sebacina* (9.24%), Nectriaceae (8.35%), *Russula* (6.13%), *Penicillium* (4.81%), *Mortierella* (4.67%), *Amphinema* (3.55%), *Hyaloscyphaceae* (3.52%), and *Thelephoraceae* (2.68%; Figure 3). Overall, the cultivation of Sanqi resulted in an increase in the abundance of *Xanthobacteraceae*, *AD3*, *Acidobacteriales*, *Trichophaea*, Nectriaceae, and *Russula* in the PaS and PkS soils. By contrast, the abundance of *Bradyrhizobium*, *Saitozyma*, *Penicillium*, *Hyaloscyphaceae*, *Thelephoraceae*, and *Vicinamibacterales* decreased. Additionally, the abundance of *IMCC26256*, *Gaiellales*, *Mortierella*, and *Amphinema* significantly increased while that of *Elsterales*, *Subgroup_2*, *Acidothermus*, and *Sebacina* significantly decreased in the PaS soil compared to that observed in the PkS soil.

RDA was employed for investigating the correlations between soil factors and microbial communities at the level of genera. The results revealed that the bacterial and fungal community was significantly impacted by the content of TK (r^2^ = 0.8662, *p* = 0.002) and TN (r^2^ = 0.4125, *p* = 0.0055) in the Pa soil and by pH (r^2^ = 0.8778, *p* = 0.001) and NO_3_^−^–N (r^2^ = 0.959, *p* = 0.001) content in the Pk soil, WC (r^2^ = 0.8885, *p* = 0.001; r^2^ = 0.7301, *p* = 0.001) in the PaS soil and by NO_3_^−^–N (r^2^ = 0.4652, *p* = 0.001) content and SOC (r^2^ = 0.9195, *p* = 0.001) in the PkS soil (Supplementary Figure S2).

### 3.4 Analysis of bacterial and fungal network complexity and stability

**Figure 4:**
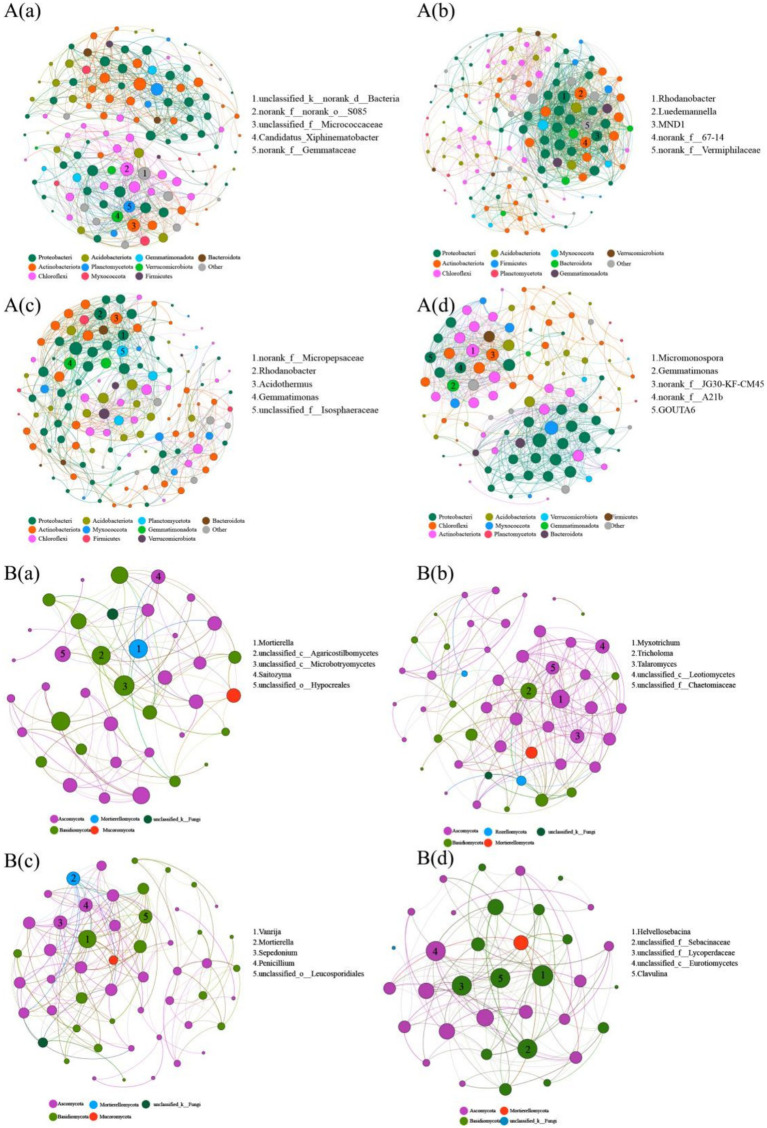
Bacterial **(A)** and fungal **(B)** network complexity in different land use systems. (a) Pa, (b) PaS, (c) Pk, and (d) PkS soils. Different colored dots denote distinct phyla to which the genera are affiliated. The figures within the nodes signify pivotal genera within the network.

**Figure 5:**
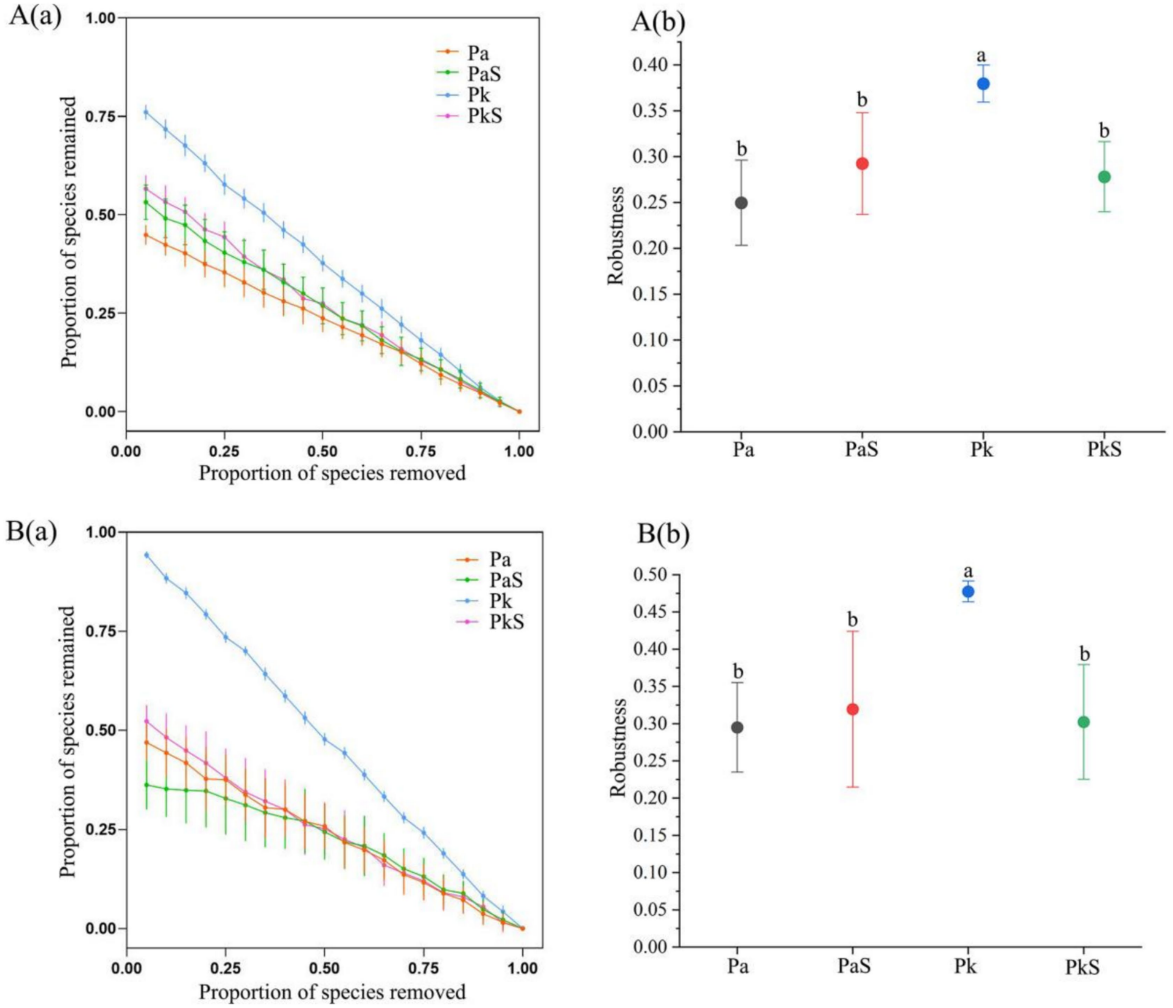
Bacterial **(A)** and fungal **(B)** network stability in different land use systems. The proportion of remaining nodes in the network after the random removal of some (a) and 50% (b) of the nodes. a and b indicate significant differences at *p* < 0.05, respectively.

Compared to that observed in the Pa soil, the complexity of the bacterial and fungal networks in the PaS soil exhibited a significant increase in the numbers of nodes and connecting edges, graph density, average degree, and average clustering coefficient, while the average path length and graph diameter exhibited a significant decrease. This indicates that the cultivation of Sanqi increased the complexity of the bacterial and fungal networks in the pine soil. Conversely, the complexity of the bacterial and fungal networks in the PkS soil was significantly reduced compared to that observed in the Pk soil (Figure 4, Supplementary Table S6). Moreover, the bacterial and fungal networks in the PaS soil exhibited good stability, with the highest stability observed in the rhizosphere soil following Sanqi cultivation. By contrast, the stability of the bacterial and fungal networks declined in the PkS soil, although the differences between the rhizosphere and bulk soils were not significant (Figure 5; Supplementary Table S7).

### 3.5 Soil metabolomics analysis

Non-targeted metabolomics techniques were employed for determining the types and contents of metabolites in the various soil samples. A total of 602 metabolites were identified across all soil samples, including lipid and lipid-like molecules (48.0%), organic acids and derivatives (7.3%), organic heterocyclic compounds (6.8%), phenylpropanoids and polyketides (6.1%), organic oxygen compounds (5.1%), aromatic compounds (5.0%), nucleosides, nucleotides, and their analogs (2.6%), organic nitrogen compounds (0.8%), hydrocarbons (0.3%), lignans, neolignans, and related compounds (0.2%), as well as homonuclear nonmetallic compounds (0.2%). Among these, the content of 223 and 379 metabolites exhibited an increase and decrease, respectively, in the PaS soil. Similarly, the content of 257 and 345 metabolites displayed an increase and decrease, respectively, in the PkS soil.

**Figure 6:**
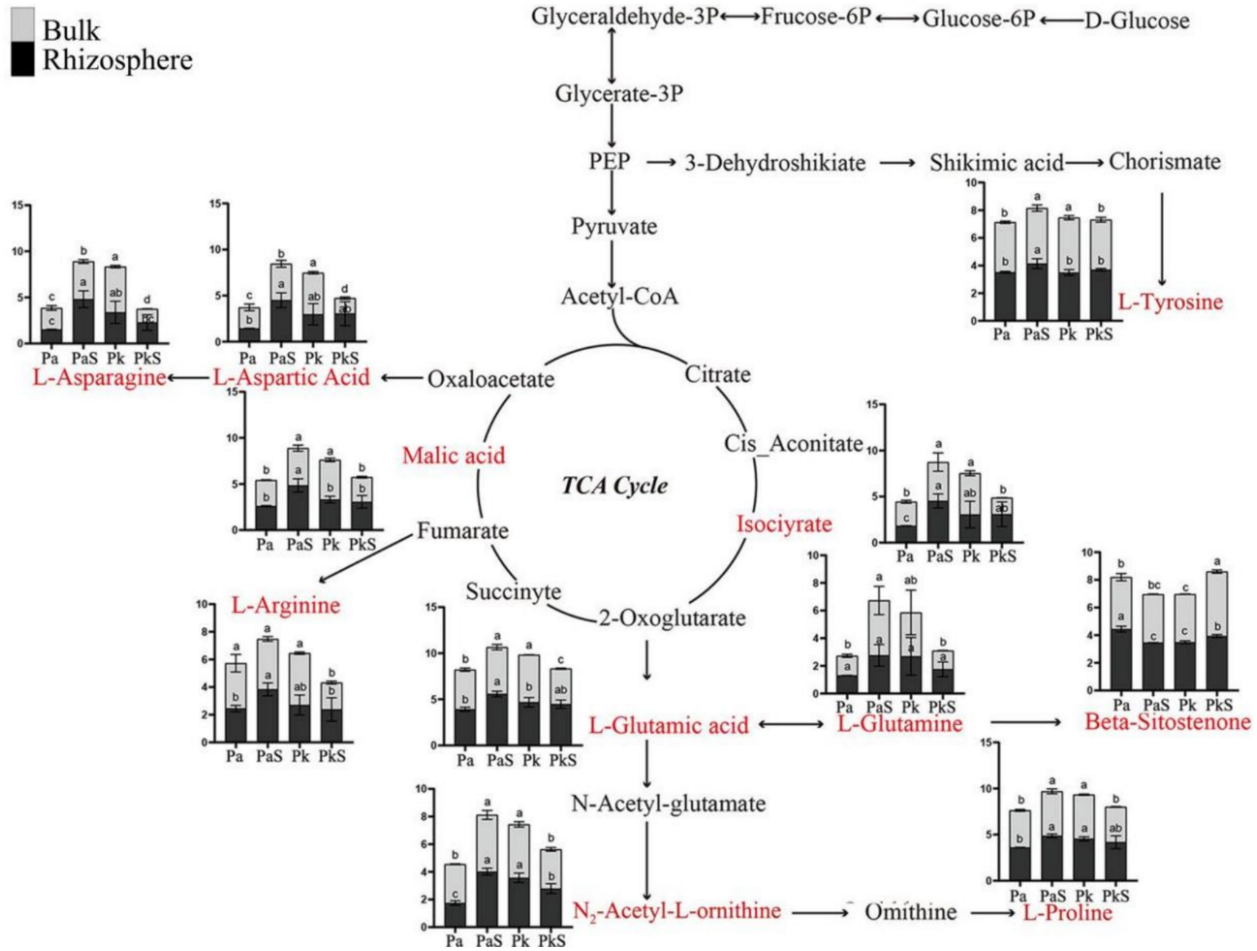
Identification of 11 differential metabolites and the associated metabolic pathways. a, b, and c indicate significant differences at *p* < 0.05, respectively.

Principal Component Analysis and volcano plot analysis revealed a significant impact of Sanqi cultivation on soil metabolites across various types of pine trees (Supplementary Figure S3). KEGG pathway enrichment analysis indicated that these differential metabolites were significantly enriched in multiple pathways, including ABC transporters, protein digestion and absorption, central carbon metabolism in cancer, biosynthesis of plant secondary metabolites, aminoacyl-tRNA biosynthesis, arginine biosynthesis, alanine, aspartate, and glutamate metabolism, and proximal tubular bicarbonate reclamation (Supplementary Figure S4). Furthermore, the following differential metabolites with roles in metabolic pathways were identified: L-glutamic acid (C_5_H_9_NO_4_), L-aspartic acid (C_4_H_7_NO_4_), L-glutamine (C_5_H_10_N_2_O_3_), L-asparagine (C_4_H_8_N_2_O_3_), L-arginine (C_6_H_14_N_4_O_2_), L-proline (C_5_H_9_NO_2_), malic acid (C_4_H_6_O_5_), beta-sitostenone (C_29_H_48_O), isocitrate (C_6_H_8_O_7_), L-tyrosine (C_9_H_11_NO_3_), and N_2_-acetyl-L-ornithine (C_7_H_14_N_2_O_3_; Supplementary Figure S5). Additionally, a metabolic pathway network comprising 11 differential metabolites was constructed herein (Figure 6). In summary, the levels of eight metabolites (L-aspartic acid, L-asparagine, L-tyrosine, malic acid, isocitric acid, L-proline, N_2_-acetyl-L-ornithine, and L-glutamic acid) exhibited a significant upward trend in the PaS soil, while that of beta-sitostenone displayed a marked decrease. Moreover, these differential metabolites exhibited positive correlation with the abundance of *Gaiellales* and *Thelephoraceae*, but negative correlation with that of *Penicillium* and *Mortierella* (Supplementary Figure S6).

### 3.6 SEM analysis

**Figure 7:**
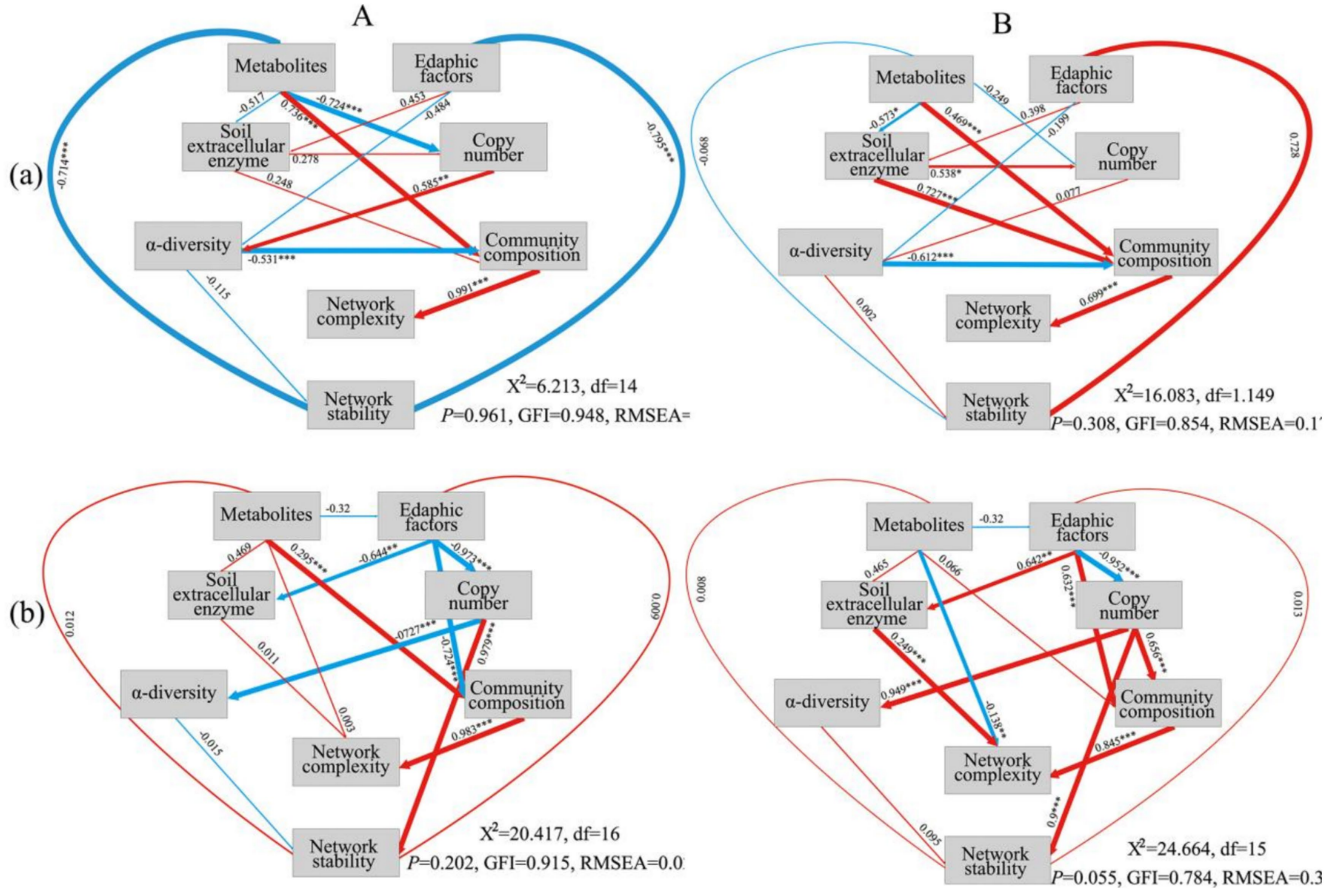
SEM analysis for soil bacteria **(A)** and fungi **(B)**. Blue: negative correlation. Red: positive correlation. (a): Pa soil. (b) PaS soil.

The results of SEM analysis revealed the direct and indirect factors that impact the complexity and stability of the bacterial and fungal networks in Pa and PaS soils. The physicochemical characteristics of the soil can further affect the complexity of the networks via effects on microbial community composition. Notably, soil enzymes and metabolites can also directly impact the complexity of the bacterial and fungal networks. Additionally, the abundance of bacteria and fungi in the PaS and Pa soils has a significant bearing on the stability of the corresponding microbial networks (Figure 7).

## 4 Discussion

### 4.1 Sanqi cultivation affects the abundance and *α*-diversity of bacteria and fungi in the PaS and PkS soils

The microbiome serves as a critical indicator of the healthy development of Sanqi–pine agroforestry systems. Previous research has revealed that the transformation of monoculture pine forests to Sanqi–pine agroforestry systems causes significant alterations in the diversity rather than community structure of pine-associated fungi (Jia et al., 2022). However, the current study revealed that the cultivation of Sanqi significantly reduced the abundance and α-diversity of bacteria and fungi in the soil of PkS rather than PaS, which is inconsistent with the results of previous studies. As reported previously, the abundance and diversity of soil microbes can remain unchanged (for instance, in the walnut–tea system), increase (mulberry–peanut system), or decrease (ginkgo–fir system) following the conversion of the land use pattern to agroforestry systems (Guo et al., 2021; Bai et al., 2022; Li M. N. et al., 2022). The factors responsible for the variations in microbial community dynamics across diverse agroforestry systems are primarily ascribed to the soil environment (Zou et al., 2023), the introduction of plant species (Mortimer et al., 2015), and the tree species involved (Xu et al., 2023). These factors exert a substantial influence on the abundance and α-diversity of soil microbes. For instance, the microbial abundance and α-diversity in the Pk soil exhibited positive correlation with the content of TN and NH_4_^+^–N but a significant negative correlation with SOC (Supplementary Table S8). Previous studies have indicated that the increase in microbial diversity is significantly influenced by an increase in SOC (wheat–nut agroforestry system) and the content of NH_4_^+^–N (Sanqi-pine agroforestry system) and TN (peanut–millet intercropping system) (Yang et al., 2023; Liu et al., 2024; Rui et al., 2024). This is because elevated levels of SOC, NH_4_^+^–N, and TN provide essential nutrients for soil microorganisms, thereby directly stimulating their growth and reproduction (Jia et al., 2024; Chandan et al., 2025). Therefore, we speculate that the decrease in NH_4_^+^–N and TN levels led to a reduction in microbial abundance and α-diversity in PkS soil.

### 4.2 Sanqi cultivation reduces the *β*-diversity of fungi in the soil of P. Kesiya rather than *P. armandii* soil

The cultivation of Sanqi significantly affects the β-diversity of fungi rather than bacteria in the pine soil and is mainly influenced by the pine species followed by the cultivation of Sanqi. These findings are in line with those of previous studies, which also emphasized the significant impact of ecological niches, Sanqi cultivation, and pine genotypes on the pine-associated microbial communities (Jia et al., 2022). Nevertheless, the cultivation of Sanqi resulted in a substantial reduction in β-diversity in the soil of *P. kesiya* rather than *P. armandii*, which may be attributed to variance in the pine species, climate, and soil characteristics. In the Sanqi–pine agroforestry systems, the genotype of the pine tree significantly influences the community structure of fungi (Jia et al., 2022); this is because pine typically forms ectomycorrhizal symbiotic associations with various fungi, whose characteristics are largely determined by the genotype of the pine tree (Peršoh, 2013; Huo et al., 2023). The results of RDA revealed that the microbial community was predominantly influenced by the content of WC in the PaS soil and by NO_3_^−^–N content and SOC in the PkS soil. The soil water content has been shown to exhibit correlation with the microbial community structure (Bao et al., 2020). Additionally, SOC has been shown to exert a substantial impact on fungal diversity, as it is one of the key factors driving the evolution of the fungal community structure (Feng et al., 2025). The cultivation of Sanqi has been postulated to influence the response of the soil microbial community to SOC and NO_3_^−^–N content in *P. kesiya* forests. This influence is likely to be intricately linked to the growth attributes of the pine trees, their root exudates, and their adaptability to the soil milieu (He et al., 2025). Therefore, SOC and NO_3_^−^–N content are pivotal factors that contribute to the decline in the diversity of soil fungal communities in *P. kesiya* forests.

### 4.3 Sanqi cultivation enhances the abundance of beneficial microorganisms and their network complexity in the soil of *P. armandii*

The main bacterial phyla in the soil of pine trees were determined to be Proteobacteria, Actinobacteria, Acidobacteriota, and Chloroflexi, while the main fungal phyla were Ascomycota, Basidiomycota, and Mortierellomycota. This finding is consistent with those of a previous study (Jia et al., 2022). The cultivation of Sanqi significantly increased the abundance of Ascomycota, Mortierellomycota (notably *Mortierella*), Gaiellales, and *Amphinema* in the soil of *P. armandii*; these fungi are responsible for the decomposition of lignin and organic matter in the soil (Zhong et al., 2022), breakdown of carbohydrates and polysaccharides (Fu et al., 2020), increase in soil nutrient content and enzyme activity (Wang et al., 2024), and nitrogen fixation (Jörgensen et al., 2024). Therefore, the increased abundance of these beneficial microorganisms is conducive to improving the ecological environment of the soil in *P. armandii*. This stable microbial ecosystem can also better provide suitable soil conditions for the growth of Sanqi, forming a virtuous ecological cycle.

The cultivation of Sanqi significantly enhanced the complexity of the microbial network in the soil of *P. armandii*, which is attributable to the direct effects of factors such as soil metabolites and extracellular enzymes on microbial complexity. Moreover, edaphic factors can indirectly influence the complexity of the microbial network by affecting the microbial community. Soil metabolites have a direct impact on the diversity and community structure of bacteria and fungi in the soil in which Sanqi is cultivated (Hei et al., 2024). This is because soil metabolites may exert inhibitory or promoting effects on microorganisms, thereby affecting microbial community dynamics (Sui et al., 2023). Extracellular enzymes in the soil are key indicators of soil quality and the metabolic activities of soil microbes, with changes in the enzyme activities directly reflecting alterations in soil ecology. Previous studies have shown that Sanqi cultivation reduces the activities of extracellular enzymes and alleviates carbon limitation for soil microbes in the soil of *P. armandii* (Rui et al., 2025). Furthermore, changes in edaphic factors can affect not only the growth rate of microbes but also alter the types of soil metabolites, thereby influencing the structure of soil microbial communities (Cao et al., 2019; Sui et al., 2023). Previous studies have revealed that Sanqi cultivation has a significant impact on soil characteristics in the SPA system (Rui et al., 2024, 2025), which is consistent with the findings of the current study. Nevertheless, variations in edaphic factors in the SPA and SPK agroforestry systems may be a contributing factor for the increased complexity of the microbial network in the soil of *P. armandii*. In Pa and PaS soils, microbial communities have a direct impact on the complexity of the microbial network. This occurs because alterations in the microbial community engender shifts in the interactions among microbes, which subsequently modify the topological structure of the microbial network (Dong et al., 2024). Furthermore, the cultivation of Sanqi significantly reduced the stability of the microbial network in PkS rather than PaS soil, which is attributable to the direct impact of the abundance of various bacteria and fungi on the stability of the microbial network. The abundance of soil microbes can promote aggregate formation and improve soil structure, thereby enhancing the stability of the microbial network (Feng et al., 2023). The cultivation of Sanqi resulted in a decrease in the abundance of bacteria and fungi in the PkS soil, with no alterations observed in the PaS soil; this may explain the reduced stability of the microbial network in the PkS soil.

### 4.4 Sanqi cultivation enhances the concentration of DAMs in the rhizosphere and bulk soils associated with *P. armandii*

The metabolites found in pine soil were primarily lipids (48.0%) followed by organic acids (7.3%), which is inconsistent with the results of previous studies (Shao et al., 2011). As per the previous study, organic (63.82 and 71.05%) and phenolic (27.80 and 16.30%) acids were the main soil metabolites found in monoculture forests of *Pinus tabuliformis* and *Ostryopsis davidiana*, respectively (Shao et al., 2011). Several factors encompassing plant species, soil environmental conditions (Wang et al., 2018), cultivation methodologies, ecological niches, and root exudates exert significant influence on the soil metabolites of pine trees (He et al., 2025). The cultivation of Sanqi may account for the elevated lipid content in PaS and PyS soils; this is because lipids are highly abundant (35.48%) in the soils associated with Sanqi cultivated in the forest understory (Hei et al., 2024) and can be transferred to the soil through various channels to stabilize soil structure and enhance microbial activity (Zhang et al., 2016; Nasir et al., 2024).

Organic acids constitute the second most significant soil metabolite and are notably abundant in PaS and PyS soils. They exert a pivotal influence on the forest soil and ecosystem, primarily due to their potential deleterious impacts on the growth of Sanqi and soil quality (Hei et al., 2023, 2024). Previous studies have revealed a disparity in the composition of organic acids in the soils associated with organically and conventionally managed Sanqi (Wu et al., 2014; Hei et al., 2023). A high concentration (>150 mg/kg) of organic acids in the soil associated with Sanqi cultivation in the forest understory can adversely impact the interplay between plant growth, soil environment, and microbial communities (Hei et al., 2023, 2024). Additionally, a considerable accumulation of heavy metal elements such as cadmium in the pine soil (Yang et al., 2023) has been shown to significantly correlate with the presence of organic acids (Wang et al., 2022). These observations have prompted the hypothesis that pine roots exude organic acids and the organic acids originating from Sanqi may also be transferred to the pine soil, which explains the higher organic acid content of the pine soil. Additionally, organic acids are capable of modulating soil metabolites via interactions with other organic and phenolic acids (Shen et al., 2020; Hei et al., 2024). They also indirectly influence soil metabolites by shaping the structure of the microbial community (Zhou et al., 2023) and engaging in nutrient cycling (Khangaroat et al., 2020). In essence, investigating the distribution patterns of lipids and organic acids in the soil of the Sanqi–pine agroforestry systems is expected to facilitate an in-depth understanding of the intricate relationship between Sanqi cultivation and the ecological dynamics of pine tree soil. In turn, this is expected to provide a scientific foundation for refining the management strategies of agroforestry systems.

The metabolic network of differential metabolites is predominantly centered on the tricarboxylic acid (TCA) cycle. Notably, the content of eight differential metabolites was substantially increased in the rhizosphere and bulk soils of PaS but significantly reduced in the PkS soil. Previous studies have shown that the metabolites in Sanqi-associated soil are affected by the cultivation patterns (He et al., 2025), which is consistent with the results obtained herein. The TCA cycle is the central pathway of cellular energy metabolism (Sultana and McClure, 2023), and the results obtained herein may indicate heightened efficiency of energy metabolism and enhanced environmental adaptability in *P. armandii* upon cocultivation with Sanqi. The differential metabolites identified herein play a crucial role in the soil ecosystem. For instance, L-glutamate and N-acetylcholine are involved in the urea cycle (Meena et al., 2019; Wang et al., 2020), while L-aspartate and L-asparagine participate in the nitrogen cycle (Patra et al., 2017). Moreover, L-tyrosine can enhance the rate of decomposition of organic matter in the soil (Wang et al., 2022). Furthermore, malate and isocitrate can regulate soil pH and promote phosphorus uptake by plants (Wang et al., 2022). Additionally, the current study revealed higher concentrations of most soil metabolites in the rhizosphere soil of *P. armandii* compared to that in the bulk soil, suggesting that the metabolites secreted by plant roots may exert a dominant influence on the composition of metabolites in the rhizosphere soil (Zhang et al., 2020). This observation aligns with the outcomes of preceding research endeavors (Liu et al., 2023). This is because the microbes occupying different ecological niches can utilize different energy sources, which affects the concentration of metabolites (Bajić et al., 2021). Correlation analysis indicated that these differential metabolites were positively correlated with beneficial microbes (such as members of Gaiellales and Thelephoraceae) and negatively correlated with pathogenic microbes (such as *Penicillium* and *Mortierella*; Hei et al., 2024). However, given that soil microbes are significant executors of metabolic activities in the soil, the composition of the microbial community governs the types and abundance of soil metabolites to a certain extent (Cheng et al., 2022). Therefore, regulating the structure of soil microbial communities allows the optimization of the types and abundance of soil metabolites, thereby promoting the growth and improving the quality of Sanqi.

## 5 Conclusion

The current study revealed that the cultivation of Sanqi increased the content of TP, NH_4_^+^–N, and TK as well as WC and pH in PaS soil but only that of NO_3_^−^–N and SOC in PkS soil. Moreover, the copy numbers as well as *α*- and *β*-diversity of bacteria and fungi were stably maintained in PaS soil but declined in PkS soil. PCoA analysis revealed that the tree species mainly influenced the changes in the community structure of bacteria and fungi. Additionally, microbial network complexity increased significantly in PaS but not PkS soil, while network stability was maintained. SEM analysis revealed that the combined effects of soil enzymes, metabolites, and physicochemical properties resulted in an increase in microbial network complexity in PaS soil. Further investigations revealed that the soil metabolites in PaS and PkS soils mainly comprised lipids (48.0%) and organic acids (7.3%). The content of eight differential metabolites was significantly higher in the rhizosphere and bulk soils of PaS. In summary, *P. armandii* significantly contributes to the robust growth of *P. notoginseng* and the overall sustainability of the agroforestry system. This discovery provides an important theoretical foundation for optimizing Sanqi cultivation practices, thereby improving both its quality and yield.

## Data Availability

The datasets presented in this study can be found in online repositories. The names of the repository/repositories and accession number(s) can be found at: https://www.ncbi.nlm.nih.gov/, PRJNA821648 (bacteria) and PRJNA821834 (fungi).
