# Supplementary material for: The cultivation of Panax notoginseng enhances the metabolites and microbial network complexity in the soil of Pinus armandii rather than Pinus kesiya
Source: Front Microbiol. 2025 Aug 6;16:1616266. doi: 10.3389/fmicb.2025.1616266 (PMC12364910; doi:10.3389/fmicb.2025.1616266)
Supplement: Supplementary file 1 [file Data_Sheet_1.docx]

***Supplementary materials***

**The cultivation of *Panax notoginseng* enhances the metabolites and microbial network complexity in the soil of *Pinus armandii* rather than *Pinus kesiya***

**Jingying Hei^1†^, Yue Li^1†^, Rui Rui^1†^, Noor Faisal^1^, Jiansong Peng^1^, Biao Wang^2*^, Shu Wang^1*^, Xiahong He^3*^**

^1^ College of Landscape Architecture and Horticulture, Southwest Forestry University, Kunming 650224, China. jyhei@swfu.edu.cn (J.H.); liyue_421@163.com (Y.L.); ruirui@swfu.edu.cn (R.R.); faisalnoor1144@gmail.com (N.F.); pengjiansong@swfu.edu.cn (J.P.)

^2^ Department of Biochemistry and Molecular Biology, School of Life Sciences, China Medical University, Shenyang 110122, China. wangbiao@cmu.edu.cn (B. W).

^3^ Yunnan Provincial Key Laboratory for Conservation and Utilization of In-forest Resource, Southwest Forestry University, Kunming, 650224, China. hxh@swfu.edu.cn (X.H.).

^†^These authors contributed equally to this work.

***Correspondence:**

wangbiao@cmu.edu.cn (B. W); wangshu@swfu.edu.cn (S.W.); hxh@swfu.edu.cn (X.H.)

**Supplementary Table S1.** The primers sequences of 16rRNA and ITS.

| **Target region** | **Primers** | **Sequence** | **PCR protocols** | **qPCR protocols** |
| --- | --- | --- | --- | --- |
| Bacterial V3-V4 | 338F | 5′-ACTCCTACGGGAGGCAGCAG-3′ | 95 ℃ for 3 min, 95 ℃ for 30 s, 55 ℃ for 30 s, 72 ℃for 45 s, 27 cycles, 72 ℃ for 10 min | 95 ℃ for 2 min, 95 ℃ for 30 s, 57 ℃ for 30 s, 72 ℃for 45 s, 40 cycles. |
|  | 806R | 5′-GGACTACHVGGGTWTCTAAT-3′ |  |  |
| Fungal ITS | ITS1F | 5′-CTTGGTCATTTAGAGGAAGTAA-3′ | 95 ℃ for 3 min, 95 ℃ for 30 s, 55 ℃ for 30 s, 72 ℃for 45 s, 35 cycles, 72 ℃ for 10 min | 95 ℃ for 2 min, 95 ℃ for 30 s, 53 ℃ for 30 s, 72 ℃for 45 s, 40 cycles. |
|  | ITS2R | 5′-GCTGCGTTCTTCATCGATGC-3′ |  |  |

**Supplementary Table S2.** Analysis of soil physicochemical properties under different treatments.

|  | Pa-B | Pa-R | PaS-B | PaS-R | Pk-B | Pk-R | PkS-B | PkS-R |
| --- | --- | --- | --- | --- | --- | --- | --- | --- |
| SOC(g/kg) | 18.31±0.80^ab^ | 11.83±0.61c | 7.87±0.08d | 15.68±1.24b | 11.81±2.82c | 7.89±2.81d | 19.21±1.39a | 17.94±0.44ab |
| TN(g/kg) | 0.46±0.01d | 0.60±0.01b | 0.38±0.01f | 0.44±0.00e | 0.53±0.01c | 0.65±0.01a | 0.44±0.00e | 0.47±0.00d |
| TP(g/kg) | 0.95±0.00d | 0.86±0.01e | 1.05±0.01a | 0.99±0.00c | 0.98±0.00c | 1.04±0.01a | 0.98±0.01c | 1.02±0.01b |
| NH4+-N (mg/kg) | 0.48±0.00cd | 0.43±0.01d | 6.01±0.08a | 0.50±0.00c | 0.45±0.00cd | 0.61±0.00b | 0.48±0.00cd | 0.49±0.00c |
| NO3--N (mg/kg) | 0.67±0.00e | 1.86±0.01a | 1.12±0.01d | 1.39±0.00c | 0.52±0.00h | 0.61±0.00f | 0.58±0.01g | 1.43±0.00b |
| WC (%) | 0.17±0.00e | 0.17±0.00e | 0.26±0.01a | 0.26±0.01a | 0.22±0.01c | 0.20±0.01d | 0.24±0.01b | 0.20±0.00d |
| pH | 6.03±0.02c | 5.66±0.02f | 6.1±0.00b | 6.55±0.06a | 6.07±0.03b | 5.78±0.02e | 5.64±0.01f | 5.97±0.02d |
| TK(mg/g) | 20.51±0.60f | 22.30±0.14e | 21.79±0.1e | 23.87±0.12d | 64.08±0.53a | 59.03±0.33b | 51.83±0.08c | 51.31±0.49c |

**Supplementary Table S3.** Alpha diversity of bacteria and fungi under different treatments.

|  | Bacterial Shannon | Bacterial Chao | Fungal Shannon | Fungal Chao |
| --- | --- | --- | --- | --- |
| Pa-R | 6.06±0.02a | 1594.02±10.6cde | 3.12±0.09d | 291.52±12.67b |
| Pa-B | 5.91±0.05ab | 1606.36±13.58bcd | 3.49±0.06ab | 349.43±13.93a |
| PaS-R | 5.86±0.05b | 1581.84±48.81cde | 2.5±0.12e | 354.41±13.77a |
| PaS-B | 6.0±0.1ab | 1647.81±34.06abc | 3.64±0.07a | 357.52±5.01a |
| Pk-R | 6.01±0.05ab | 1685.24±42.19a | 3.27±0.22cd | 352.83±7.41a |
| Pk-B | 5.89±0.17b | 1671.02±34.96ab | 3.3±0.08cd | 306.26±17.66b |
| PkS-R | 5.64±0.02c | 1532.39±59.52de | 3.1±0.02d | 306.42±28.64b |
| PkS-B | 5.65±0.05c | 1519.44±55.35e | 3.35±0.01bc | 213.8±9.26c |

**Supplementary Table S4.** Analysis of Similarities (ANOSIM) for bacterial and fungal communities.

|  | Bacteria | | Fungi | |
| --- | --- | --- | --- | --- |
|  | Bray-Curtis ANOSIM | *P*-value | Bray-Curtis ANOSIM | *P*-value |
| Tree species | 0.329 | 0.004 | 0.41864875 | 0.007 |
| Sanqi cultivation | 0.112 | 0.04 | 0.1971 | 0.02 |
| Rhizosphere-bulk | 0.01 | 0.78 | 0.02 | 0.56 |

**Supplementary Table S5.** Beta diversity of bacteria and fungi under different treatments.

|  | Bacteria | Fungi |
| --- | --- | --- |
| Pa-R | 0.13±0.01b | 0.13±0.04abc |
| Pa-B | 0.14±0.01b | 0.11±0.02abc |
| PaS-R | 0.15±0.04ab | 0.08±0.02c |
| PaS-B | 0.16±0.03ab | 0.11±0.02abc |
| Pk-R | 0.2±0.04a | 0.16±0.04a |
| Pk-B | 0.13±0.02b | 0.14±0.03ab |
| PkS-R | 0.16±0.04ab | 0.13±0.04abc |
| PkS-B | 0.16±0.03ab | 0.1±0.01bc |

**Supplementary Table S6.** Seven topological coefficients of the soil bacterial and fungal sub-network under different treatments.

| Treatments | Bacteria | | | | | | | Fungi | | | | | | |
| --- | --- | --- | --- | --- | --- | --- | --- | --- | --- | --- | --- | --- | --- | --- |
|  | Node number | Edge number | Graph density | Average degree | Clustering coefficient | Average path length | Graph diameter | Node number | Edge number | Graph density | Average degree | Clustering coefficient | Average path length | Graph diameter |
| PaS | 136 | 1195 | 0.13 | 17.57 | 0.55 | 3.05 | 8 | 50 | 225 | 0.18 | 9 | 0.53 | 2.63 | 6 |
| Pa | 129 | 832 | 0.1 | 12.9 | 0.49 | 3.22 | 9 | 39 | 125 | 0.17 | 6.41 | 0.49 | 3.02 | 8 |
| PkS | 108 | 723 | 0.13 | 13.39 | 0.51 | 3.31 | 10 | 35 | 131 | 0.22 | 7.49 | 0.68 | 3.46 | 8 |
| Pk | 138 | 766 | 0.08 | 11.1 | 0.49 | 3.10 | 10 | 52 | 221 | 0.16 | 8.11 | 0.6 | 2.92 | 8 |

**Supplementary Table S7.** The network stability of bacteria and fungi under different treatments.

|  | Network stability of bacteria | Network stability of fungi |
| --- | --- | --- |
| Pa-R | 0.28±0.04bc | 0.31±0.06bc |
| Pa-B | 0.22±0.04c | 0.28±0.07bc |
| PaS-R | 0.34±0.02ab | 0.39±0.05ab |
| PaS-B | 0.25±0.05c | 0.24±0.07c |
| Pk-R | 0.38±0.02a | 0.48±0.02a |
| Pk-B | 0.38±0.02a | 0.48±0.01a |
| PkS-R | 0.28±0.04bc | 0.30±0.07bc |
| PkS-B | 0.27±0.04bc | 0.3±0.09bc |

**Supplementary Table S8.** Analysis of the correlation between bacterial/fungal copy number/α diversity and physicochemical properties.

|  | SOC (g/kg) | TN (g/kg) | TP (g/kg) | NH_4_^+^-N (mg/kg) | NO_3_^-^-N (mg/kg) | WC (%) | pH | TK (mg/g) |
| --- | --- | --- | --- | --- | --- | --- | --- | --- |
| Copy number (Bacteria) | -0.236 | 0.566** | -0.268 | 0.631** | 0.696** | -0.223 | 0.161 | -0.171 |
| Copy number (Fungi) | -0.427* | 0.411* | -0.351 | 0.634** | 0.774** | -0.063 | 0.119 | -0.354 |
| α diversity (Bacteria) | -0.548** | 0.508* | 0.157 | 0.478* | 0.458* | -0.204 | 0.496* | 0.707** |
| α diversity (Fungi) | -.437* | 0.541** | 0.308 | 0.435* | 0.495* | 0.064 | 0.607** | 0.563* |


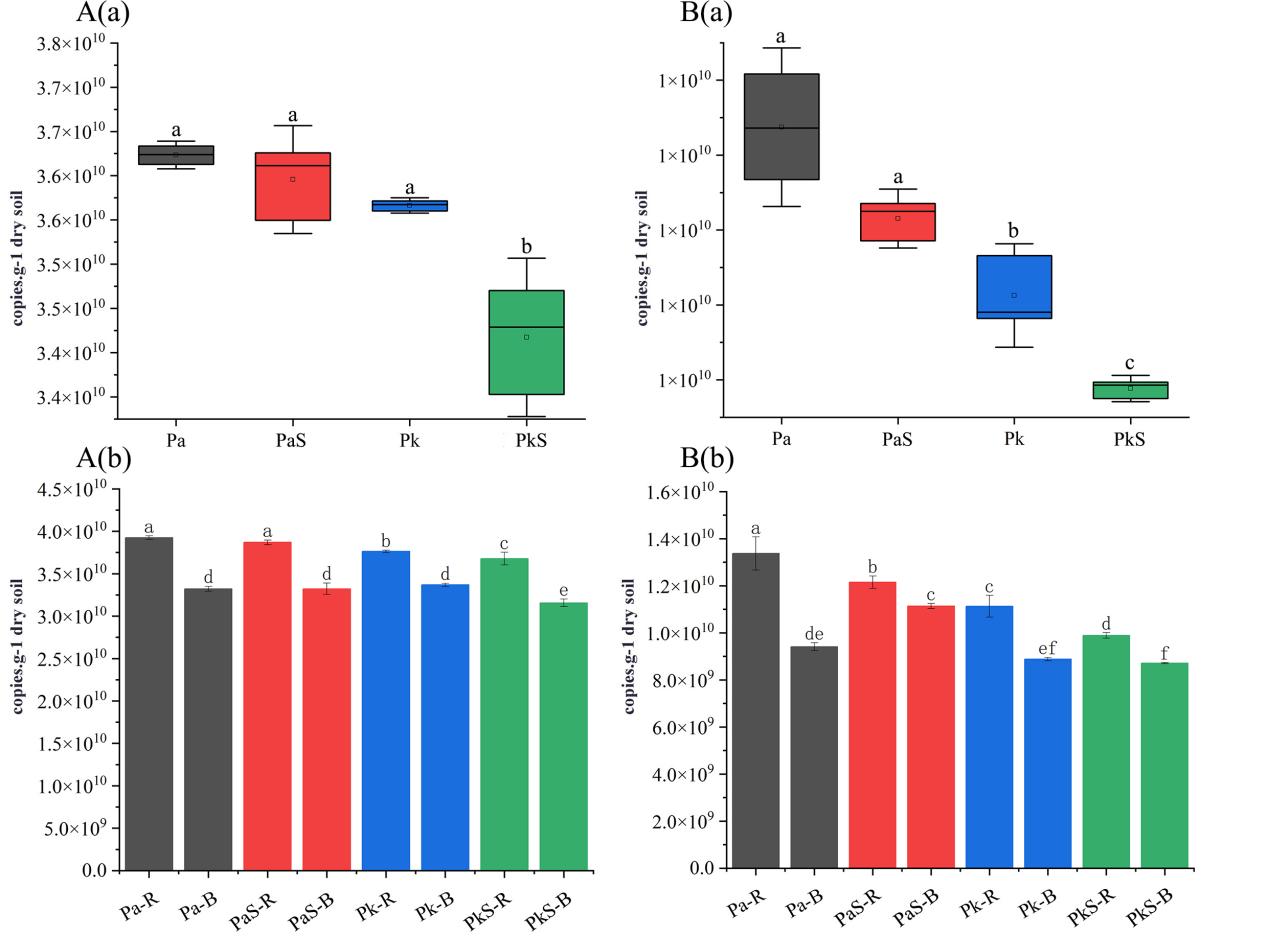


**Supplementary Figure S1.** The copy number of bacteria (A) and fungi (B) under different treatments.


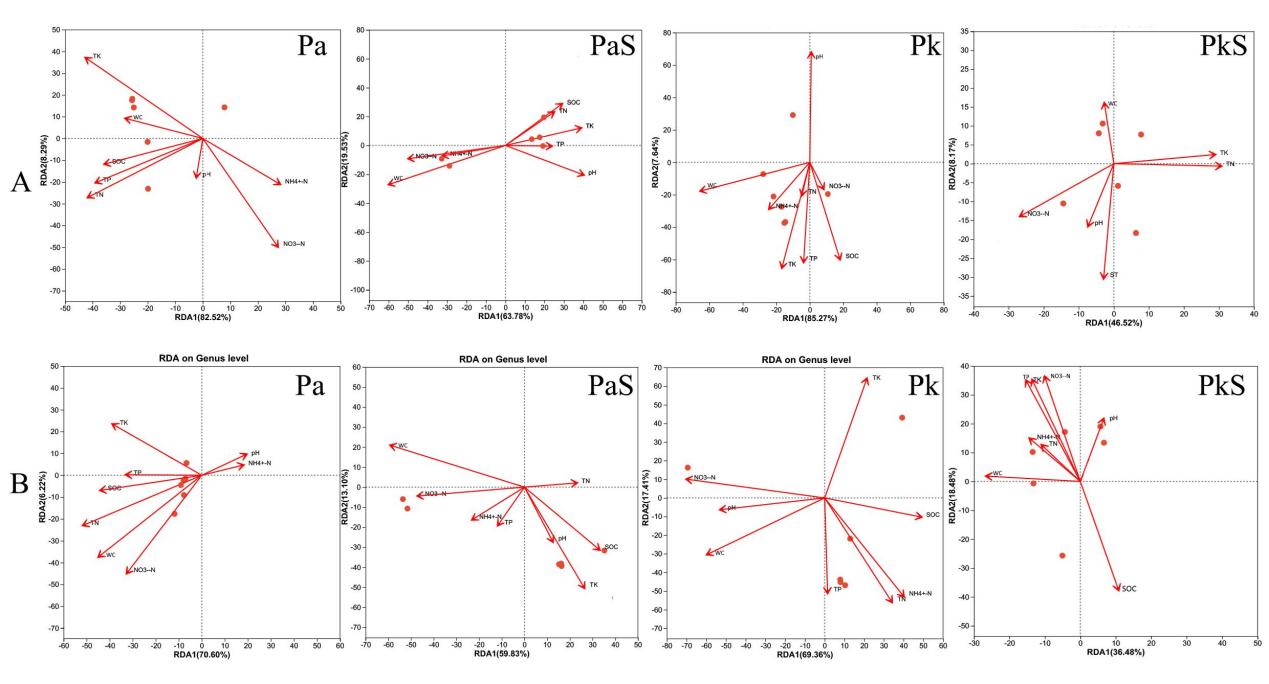


**Supplementary Figure S2.** The RDA analysis of the bacteria (A)/fungi (B).


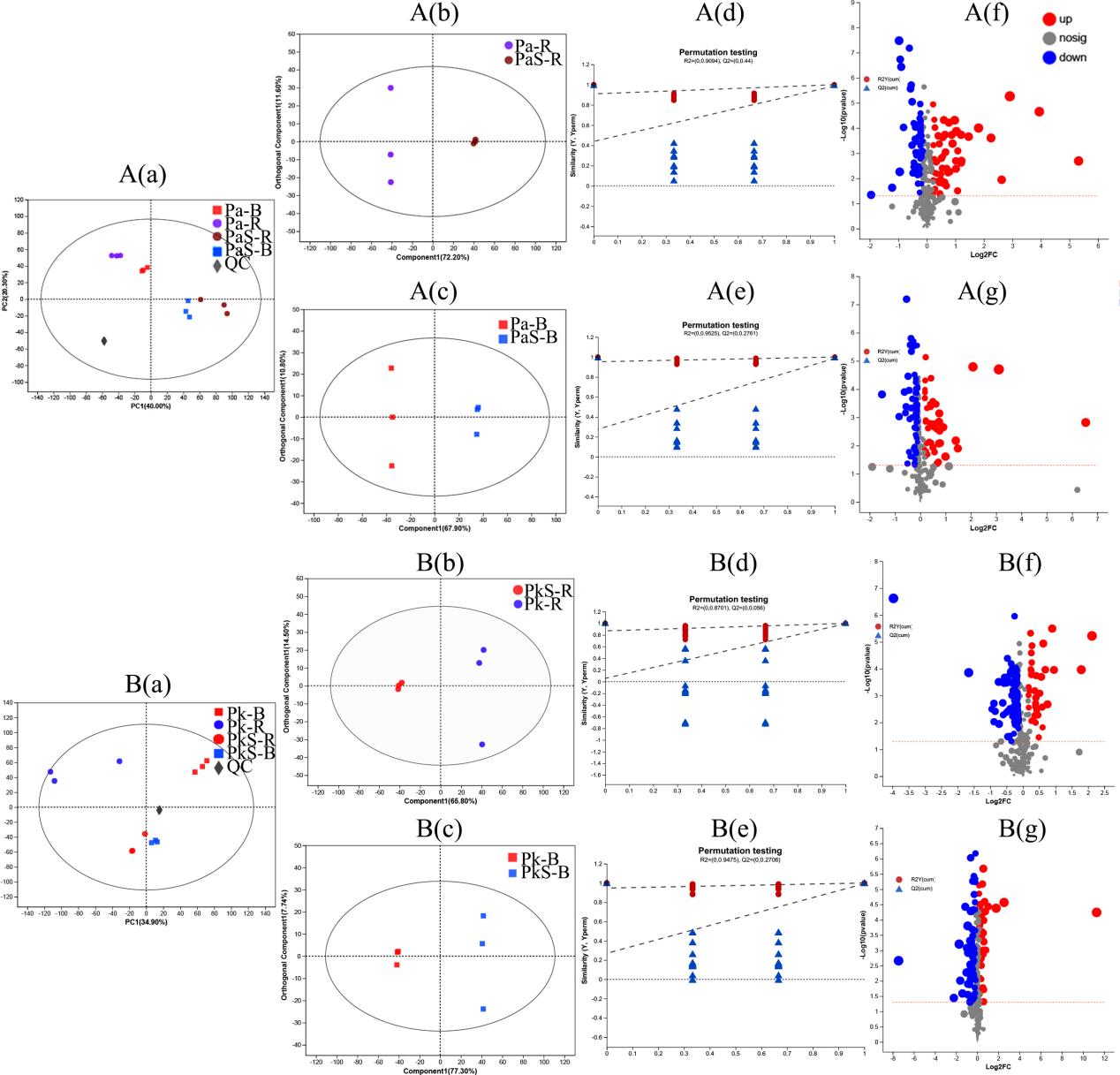


**Supplementary Figure S3.** Principal Component Analysis (PCA) and volcano map analysis.


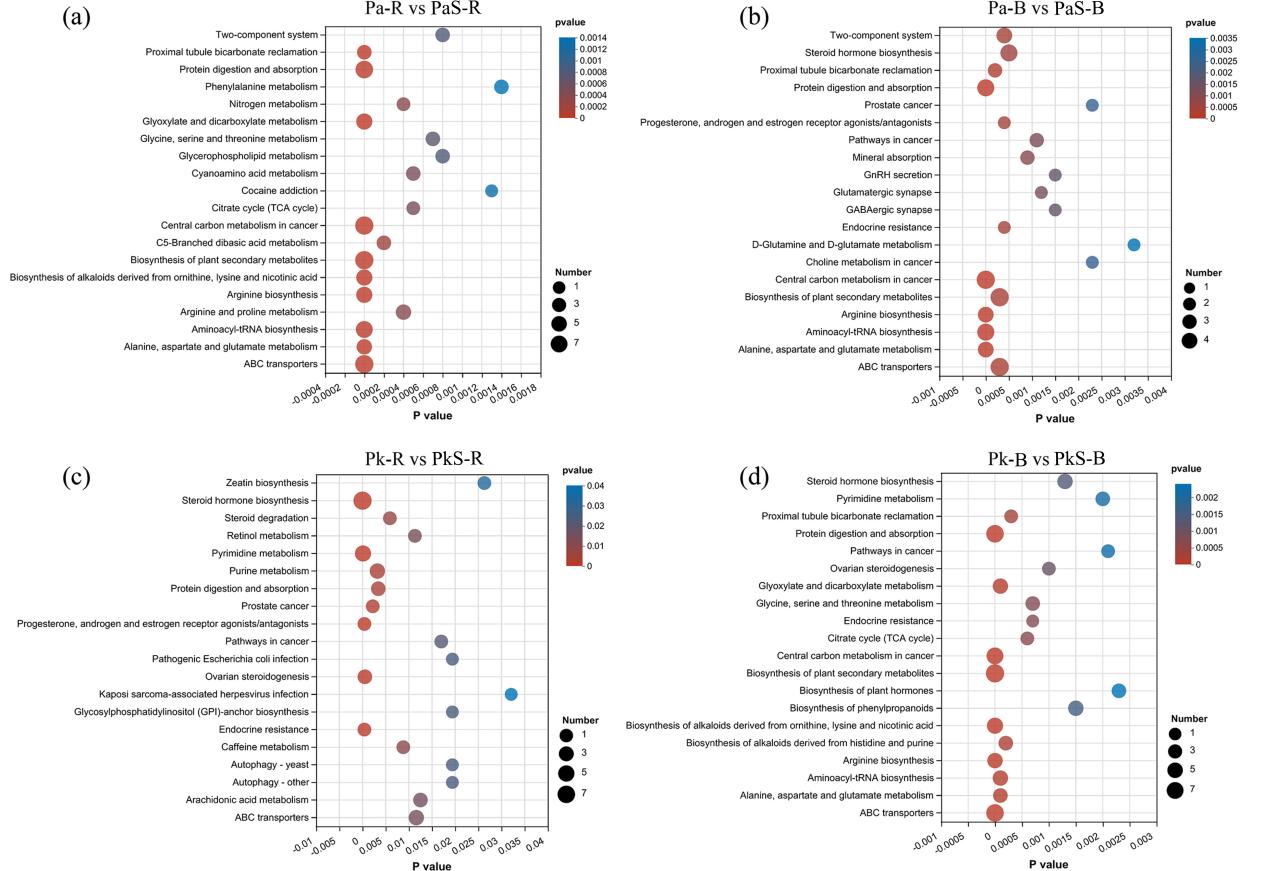


**Supplementary Figure S4.** Differential metabolites involved in metabolic pathways


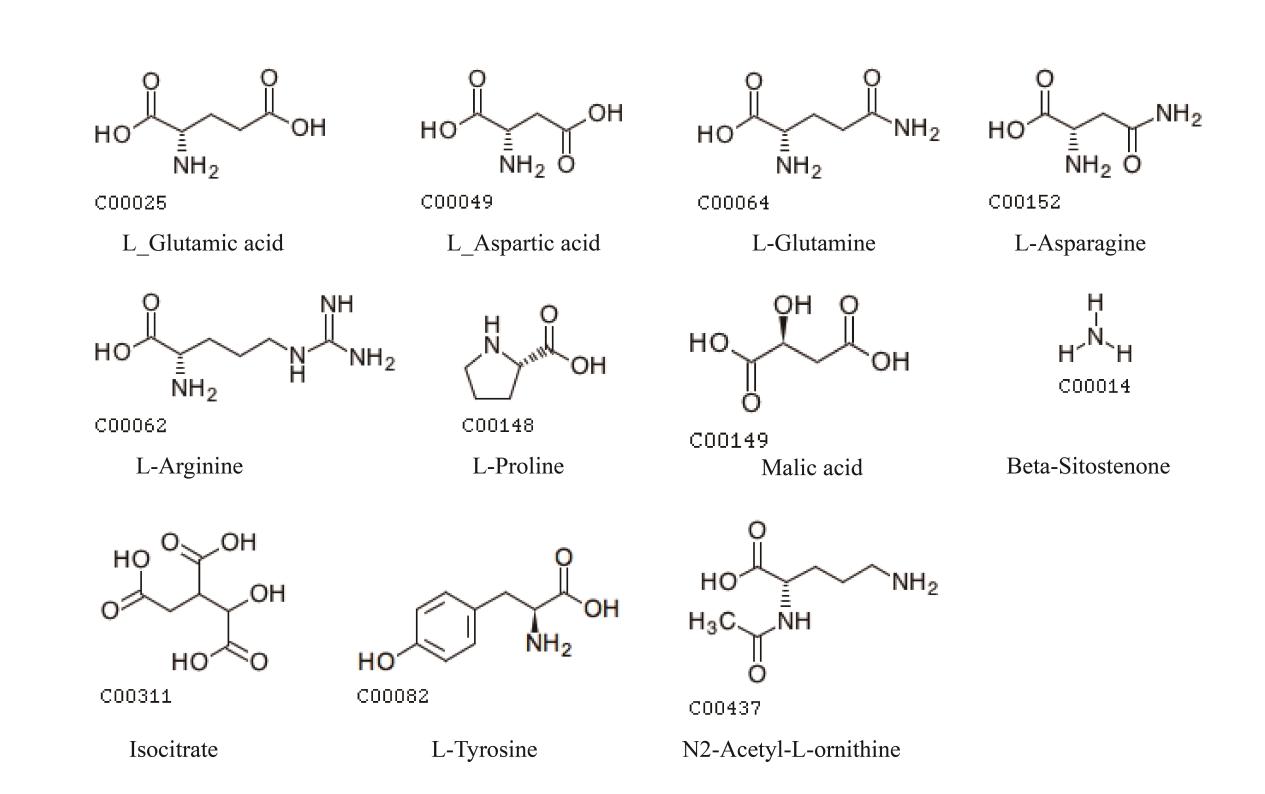


**Supplementary Figure S5.** The structural formula for 11 differential metabolites.


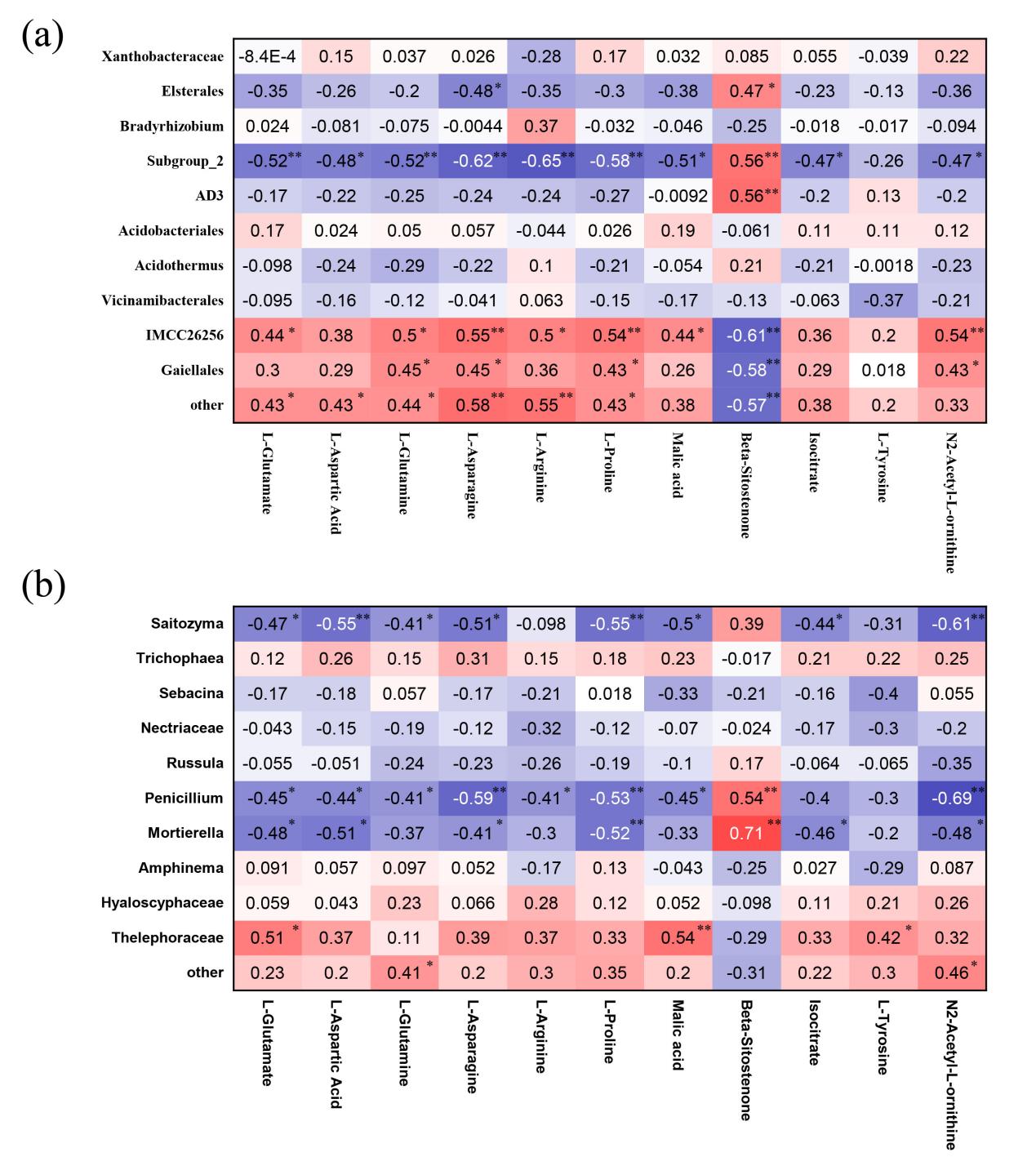


**Supplementary Figure S6.** Correlation Analysis between differential metabolites and the genera of bacteria (a) and fungi (b).
